# Carbon Fluxes in Potato (*Solanum tuberosum*) Remain Stable in Cell Cultures Exposed to Nutritional Phosphate Deficiency

**DOI:** 10.3390/biology12091190

**Published:** 2023-09-01

**Authors:** Jiang Zhou He, Sonia Dorion, Laura Michell Carmona-Rojas, Jean Rivoal

**Affiliations:** 1 Institut de Recherche en Biologie Végétale, Université de Montréal, 4101 Rue Sherbrooke Est, Montréal, QC H1X 2B2, Canada; jzh961@gmail.com (J.Z.H.); sonia.dorion@umontreal.ca (S.D.); laura.carmona@udea.edu.co (L.M.C.-R.); 2 Grupo de Biotecnologiía, Facultad de Ciencias Exactas y Naturales, Universidad de Antioquia, Medelliín 050010, Colombia

**Keywords:** plant cell culture, phosphate deficiency, glycolysis, respiration, metabolic flux

## Abstract

**Simple Summary:**

Phosphorus is an essential nutrient for plants. It is usually available in the form of inorganic phosphate in the plant environment. Yet, most environments contain extremely low amounts of phosphate, causing a major nutritional deficiency for plant life. During their evolution, plants have acquired a number of adaptations that help them to survive chronic phosphate deficit. Many of these adaptations are documented in the scientific literature and show changes in gene expression and modifications in the levels of enzymes and metabolites involved in plant carbon and respiratory metabolism. This research uses potato cells cultivated in vitro to measure major metabolic carbon fluxes (rates at which molecules are processed, consumed or degraded) in response to two phosphate regimes (normal and deficiency). Our results show a remarkable stability of several metabolic fluxes in cells regardless of their phosphate regime. This is the case for the rate at which the carbon source is taken up from the medium. Two important metabolic fluxes used to fuel cell respiration are also not affected by phosphate. In all the experiments, cell age is the main factor affecting carbon metabolic fluxes. These findings lead us to conclude that potato cells maintain stable carbon fluxes during phosphate deficiency.

**Abstract:**

Nutritional phosphate deficiency is a major limitation to plant growth. Here, we monitored fluxes in pathways supporting respiratory metabolism in potato (*Solanum tuberosum*) cell cultures growing in control or limiting phosphate conditions. Sugar uptake was quantified using [U-^14^C]sucrose as precursor. Carbohydrate degradation through glycolysis and respiratory pathways was estimated using the catabolism of [U-^14^C]sucrose to ^14^CO_2_. Anaplerotic carbon flux was assessed by labeling with NaH^14^CO_3_. The data showed that these metabolic fluxes displayed distinct patterns over culture time. However, phosphate depletion had relatively little impact on the various fluxes. Sucrose uptake was higher during the first six days of culture, followed by a decline, which was steeper in Pi-sufficient cells. Anaplerotic pathway flux was more important at day three and decreased thereafter. In contrast, the flux between sucrose and CO_2_ was at a maximum in the mid-log phase of the culture, with a peak at Day 6. Metabolization of [U-^14^C]sucrose into neutral, basic and acidic fractions was also unaffected by phosphate nutrition. Hence, the well-documented changes in central metabolism enzymes activities in response to Pi deficiency do not drastically modify metabolic fluxes, but rather result in the maintenance of the carbon fluxes that support respiration.

## 1. Introduction

Phosphorus (P) is an essential macronutrient in plants. It is a crucial component of membrane phospholipids needed for cell and organelle integrity [1]. As a component of DNA and RNA, it is involved in genetic information storage and transmission [1]. P also plays an essential function in metabolism, energy metabolism in particular, due to its involvement in phosphoanhydride bonds in ATP [1,2]. P is available to plants in the form of inorganic P (orthophosphate, Pi). Given the importance of P for their nutrition, the common scarcity of Pi-containing minerals in soils often results in a nutritional stress situation for plants [1,2,3,4]. For instance, it is estimated that 70% of the world’s agricultural lands limit Pi nutrition of cultivated plants [5]. This situation has led to the massive use of P fertilizers derived from non-renewable fossil deposits to sustain agricultural productivity for animal and human use [6,7].

Plant metabolic adaptations to Pi deficiency involve a combination of mechanisms that comprise modulation of gene expression [8,9], changes in protein/enzyme steady-state levels [10,11,12] and posttranslational regulation [13,14]. A number of these responses are well characterized [5,15,16]. In particular, low nutritional Pi availability is known to elicit metabolic adjustments in plant respiratory metabolism. For example, Pi deficiency results in higher activity for enzymes involved in Pi recycling and scavenging [17,18,19] as well as induction of genes coding for Pi transporters [4]. There is also ample evidence that plants exploit built-in flexibility in their primary metabolism and enhance the activity of enzymes that conserve Pi and ATP during Pi starvation, in particular in the glycolytic pathway [19,20]. For instance, induction of phospho*enol*pyruvate carboxylase (PEPC) and/or phospho*enol*pyruvate phosphatase activities under Pi-deficient conditions has been demonstrated in various systems [20,21,22,23,24]. These two enzymes bypass the reaction of ADP-dependent pyruvate kinase and generate, respectively, oxaloacetate and pyruvate needed to supply C to the TCA cycle.

Despite these advances, some aspects of metabolic adaptation to low Pi are still not fully understood. For example, it is commonly assumed that changes in gene expression lead to modification in enzyme levels that modulate flux in metabolic pathways. In *Arabidopsis thaliana*, experiments using steady-state metabolic flux analysis (labeling of cell cultures with [^13^C]glucose coupled to metabolic modeling) reported a small increase in anaplerotic C flux [25], thereby bringing support to the view that the frequently reported PEPC induction during Pi deprivation is needed to maintain anaplerotic flux under low-Pi conditions [26,27]. Similarly, induction of pyrophosphate:fructose-6-phosphate 1-phosphotransferase (PPi-PFK), one of the markers of Pi deficiency, can be interpreted as a way to modulate carbon flow in the glycolytic pathway [28]. However, much progress remains to be achieved in order to understand whether metabolic flux adjustments occur during adaptation to Pi deprivation and whether induction of enzymes results in flux changes.

Potato (*Solanum tuberosum*) is one of the most important staple food crops worldwide [29,30]. Its raising global production is heavily dependent on the use of Pi fertilizer [30]. In particular, Pi affects potato tuber yield and tuber set [31]. Given the major importance of Pi in metabolism, it is thus essential to expand our understanding of the impact of Pi deficiency on C fluxes in potato. In a previous study, we addressed Pi nutrition in potato cell cultures [24]. We surveyed the activities of a large number of glycolytic and respiratory enzymes and the level of key primary metabolites in Pi-sufficient (+Pi) and Pi-deficient (−Pi) conditions [24]. In the present study, we used the same experimental system to investigate the metabolism of [U-^14^C]sucrose (Suc) and NaH^14^CO_3_ in order to test the hypothesis that changes in central metabolic fluxes occur in response to modifications in Pi nutrition. These two radioactive compounds can be used as tracers to quantify the metabolic fluxes that use them as substrates or precursors. Our data show that C fluxes in potato cell cultures are relatively stable under Pi deficiency. Our results suggest that the magnitude of carbon fluxes in potato cell cultures is more influenced by culture age than nutritional Pi status.

## 2. Materials and Methods

### 2.1. Chemicals and Plant Material

All cell culture supplies and chemicals used in this study were of analytical grade and purchased from Sigma-Aldrich Canada (Oakville, ON, Canada) or Fisher Scientific (Ottawa, ON, Canada). Moravek (Brea, CA, USA) was the supplier of [U-^14^C]Suc and NaH^14^CO_3_ (respective catalog numbers MC266 and MC208). Chromatographic resins (Dowex AG 50W-X8 (H^+^) and Dowex AG 1-X8 (formate)) were obtained from Bio-Rad Laboratories (Mississauga, ON, Canada). Potato heterotrophic cell cultures established in our laboratory [32] were subcultured every 14 days and grown in a Murashige and Skoog (MS) medium [33], exactly as described previously for +Pi (inoculated with 2.5 mM Pi) or −Pi (inoculated with 125 µM Pi) conditions [24]. Cells were grown on a gyratory shaker at 140 rpm at 22 °C. They were harvested by centrifugation (4500× *g* for 10 min) at different times of the culture cycle and further used for labeling studies.

### 2.2. Isotopic Labeling Experiments

For labeling with [U-^14^C]Suc, pelleted cells were resuspended in an MS medium adjusted to the concentrations of Suc and Pi present before centrifugation for each of the time points under study. For this, medium concentrations of Suc were determined by HPLC [34], and Pi was determined using a spectrophotometric assay [24]. The resuspended cells were also rapidly adjusted to an FW to volume ratio of approximately 100 mg/mL. The exact ratio of FW/mL was determined subsequently by vacuum filtration over Whatman #1 filter paper using triplicate 10 mL samples of the resuspended cultures. Two mL of cells (~200 mg FW) were transferred to the external compartment of a 125 mL Warburg flask containing the [U-^14^C]Suc tracer at a specific radioactivity of 2.64 × 10^3^ Bq µmol^−^^1^. The center well of the flask received 2 mL of 1M KOH and the flask was immediately closed with a sleeve rubber stopper. Incubation was performed on a gyratory shaker at 120 rpm for 6 h. At the end of the experiment, the cell suspension was pipetted into the barrel of a disposable column (Thermo Scientific catalog number 1138750) inserted in a 50 mL Falcon tube. This allowed quick separation of the cells from the medium by centrifugation (4500× *g* for 5 min). The cells, collected on the filter at the base of the column, were recovered by a second centrifugation with the column oriented top-down in another tube. The biological material was then subjected to ethanol extraction followed by chromatographic separation on two 3 mL columns (Dowex AG 50W-X8 (H+) and Dowex AG 1-X8 (formate)) arranged in tandem [35]. This generated neutral, anionic and cationic fractions, which were assayed for radioactivity using a scintillation counter as previously described [34]. [U-^14^C]Suc metabolized to ^14^CO_2_ was determined from the radioactivity trapped in KOH [36]. Uptaken radioactivity was calculated from the tracer recovered in cells and in the CO_2_ fraction. Recovery of the radioactive label was >92%. In all the experiments, the specific radioactivity of the supplied [U-^14^C]Suc was used to calculate the molar fluxes of carbon [37].

The protocol for labeling with NaH^14^CO_3_ was adapted from a previous procedure [38]. Cells were harvested by centrifugation as described above and resuspended at a precisely determined FW/V ratio of about 12.5 mg/mL of the MS medium containing 20 mM Hepes pH 7.3 with high or low Pi. Four mL of this cell suspension were then transferred to the external compartment of a Warburg flask. One hundred and twenty µL of a NaH^14^CO_3_ tracer (85.4 × 10^3^ Bq with a specific radioactivity of 3.55 × 10^4^ Bq µmol^−^^1^) previously diluted in a 200 mM Potassium bicarbonate/13.3 mM Hepes-KOH (pH 7.3) were added to the cell suspension and the flask was immediately closed with a sleeve type rubber stopper. Incubation with the tracer was performed on a gyratory shaker at 140 rpm. After 10 min of labelling, 10 mL of 0.5 N HCl made in 75% (*v*/*v*) ethanol was injected into the cell suspension through the rubber cap. The center well of the flask received 2 mL 1 M KOH, and the flask was kept on the gyratory shaker for an additional 3 h. In preliminary trials, these conditions were found to be sufficient to immediately quench the metabolism of the tracer in the cells. This treatment also completely volatilized any unincorporated NaH^14^CO_3_ present in the outer compartment of the flask and allowed its recovery in the center well containing the KOH trap. Incorporation of ^14^C in primary metabolites was used to estimate the anaplerotic flux through PEPC. This was determined from the acidic ethanol-soluble radioactivity present in the outer compartment of the Warburg flask. For this, the cell suspension was transferred into the barrel of a disposable column and processed as described above to separate soluble material from the cell debris. Tracer recovery was >95%. The specific radioactivity of the NaH^14^CO_3_ tracer was used to calculate molar fluxes of carbon.

### 2.3. Statistics

Different cell cultures were treated as independent experimental units in order to have a factorial experiment consisting of two nutritional phosphate regimes (+Pi and −Pi), four cell ages (0, 3, 6, and 9 days), and three independent biological replicates per treatment. In labeling studies designed to evaluate the Suc uptake from the medium with [U-^14^C]Suc, 12-day-old cells were also used. Statistical analyses were performed using the R programming language (version 4.2.2) employing the doebioresearch package (version 0.1.0) [39]. A two-way ANOVA test was used to determine the effect of cell ages and Pi levels on each response variable. Additionally, Fisher’s Least Significant Difference (LSD) test was used to determine statistically significant differences between specific pairs of groups with a confidence level of 95%.

## 3. Results

### 3.1. Tracer Uptake by +Pi and −Pi Cells

**Figure 1:**
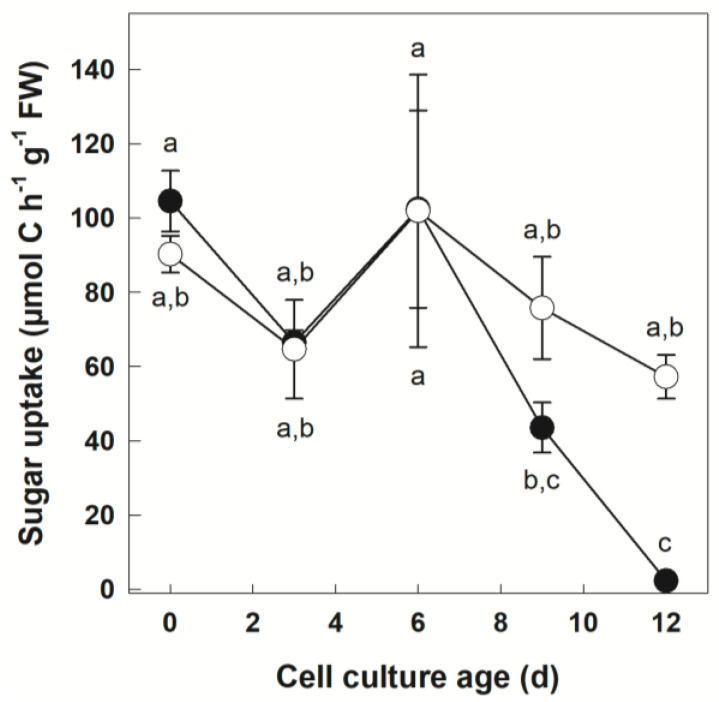
Sugar uptake as a function of culture time in +Pi and −Pi potato cells. Cells growing under two different nutritional Pi regimes were incubated with [U-^14^C]Suc. The C flux between the medium and the cells was calculated using the specific radioactivity of the tracer and the radioactivity recovered in the various cellular fractions and the KOH trap for ^14^CO_2_ following labeling. Data points represent mean ± SD (*n* = 3 biological replicates). Different letters next to the symbols (●: +Pi; ○: −Pi) indicate significant statistical differences (*p* < 0.05, Fishers LSD test).

The uptake of [U-^14^C]Suc fed to cells was assessed in cultures growing under Pi-sufficient and Pi-deficient conditions (Figure 1). The results were expressed in µmol of C taken up by cells using the specific radioactivity of Suc for calculations. 

During the culture cycle, progression of the carbon source uptake from the medium was similar in both Pi nutrient regimes. In −Pi conditions, it was around 90 µmol C h*^−^*^1^ g*^−^*^1^ FW at Day 0 and remained above 50 µmol C h*^−^*^1^ g*^−^*^1^ FW for the whole duration of the culture cycle. The results were similar for the +Pi culture until Day 6. At Day 9, C uptake sharply declined in the +Pi culture. At Day 12, the uptake of extracellular sugar was different between the two cultures, with extremely low values in the +Pi culture. Because of the lack of significant sugar uptake at this time point for the +Pi culture, we did not characterize C fluxes beyond Day 9 of culture in subsequent experiments.

### 3.2. Metabolization of [U-^14^C]Suc by +Pi and −Pi Cells

**Figure 2:**
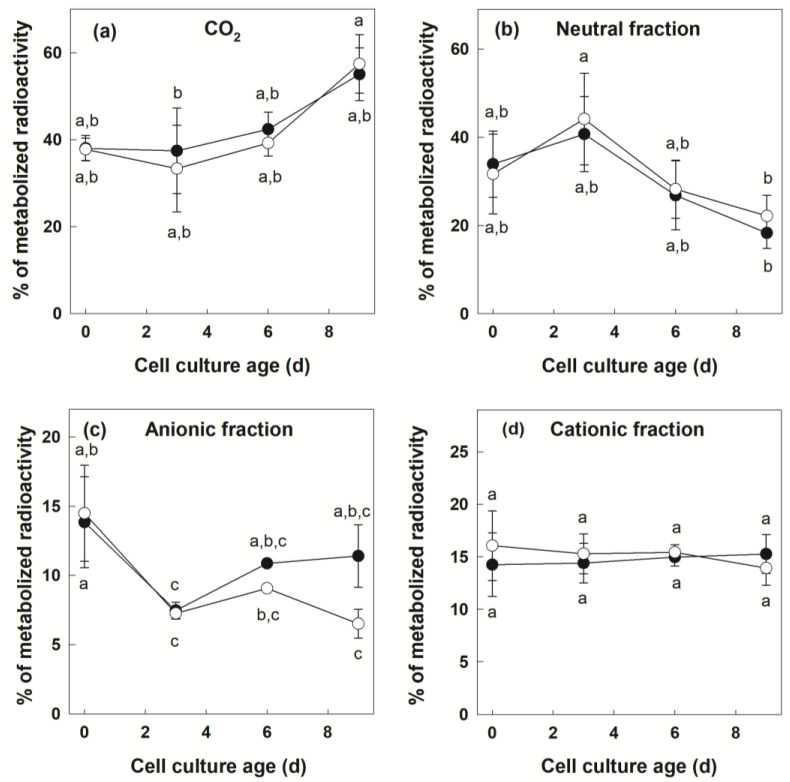
Distribution of ^14^C metabolized in volatile and soluble fractions during labeling with [U-^14^C]Suc as a function of culture time in +Pi and −Pi potato cells. Cells growing under Pi-sufficient and Pi-deficient regimes were labeled for 6 h with [U-^14^C]Suc at various time points during the culture. The metabolized radioactivity was recovered in various fractions at the end of the labeling. The distribution of ^14^C recovered in (**a**) CO_2_, (**b**) neutral, (**c**) anionic and (**d**) cationic fractions is expressed as a percentage of total recovered metabolized radioactivity. Data points represent mean ± SD (*n* = 3 biological replicates). When error bars are not visible, they are smaller than the symbol. Different letters next to symbols (●: +Pi; ○: −Pi) indicate significant statistical differences (*p* < 0.05, Fishers LSD test).

We followed the metabolic fate of supplied [U-^14^C]Suc between Days 0 and 9 of the culture cycle in +Pi and −Pi cells (Figure 2). 

Once the radioactive tracer was taken up by cells, its metabolic use could be quantified by accounting for radioactivity present in different fractions at the end of the experiment. Radioactivity present in CO_2_ is indicative of Suc used for catabolic oxidation by the glycolytic pathway and the tricarboxylic acid cycle. Fractionation of ethanol-soluble metabolites into neutral, anionic (acidic) and cationic (basic) fractions is indicative of the labeling of pools of free sugars, organic acids and amino acids, respectively. For all treatments and time points investigated, radioactivity recovered in these different fractions was expressed as a percentage of the total recovered metabolized label (Figure 2). In these experiments, at all the time points considered, there was no difference between the two cultures for the proportion of [U-^14^C]Suc oxidized to CO_2_ (Figure 2a, Fishers LSD test, *p* < 0.05). At day 0, Suc oxidation to CO_2_ accounted for a little less than 40% of metabolized Suc. Over the next days, this proportion slowly increased to reach values above 50% at Day 9. The evolution of labeling of the neutral fraction (Figure 2b) was also similar in +Pi and −Pi conditions (Figure 2b, Fishers LSD test, *p* < 0.05). The proportion of radioactivity present in this fraction was around 30% at Day 0. It increased slightly at Day 3, and decreased steadily thereafter. Labeling of the anionic compounds initially accounted for about 15% of the total radioactivity metabolized in the cells (Figure 2c). This proportion decreased slightly during the culture cycle in +Pi and −Pi conditions. Compared to Day 0, the decrease was significant for both cultures at Day 3, and for the −Pi culture at subsequent time points. However, the difference between the two Pi regimes was not significant (Fishers LSD test, *p* < 0.05). Finally, in both +Pi and −Pi cells, labeling of the cationic fraction was remarkably similar and stable throughout the culture cycle at around 15% of the total metabolized label (Figure 2d, Fishers LSD test, *p* < 0.05).

**Figure 3:**
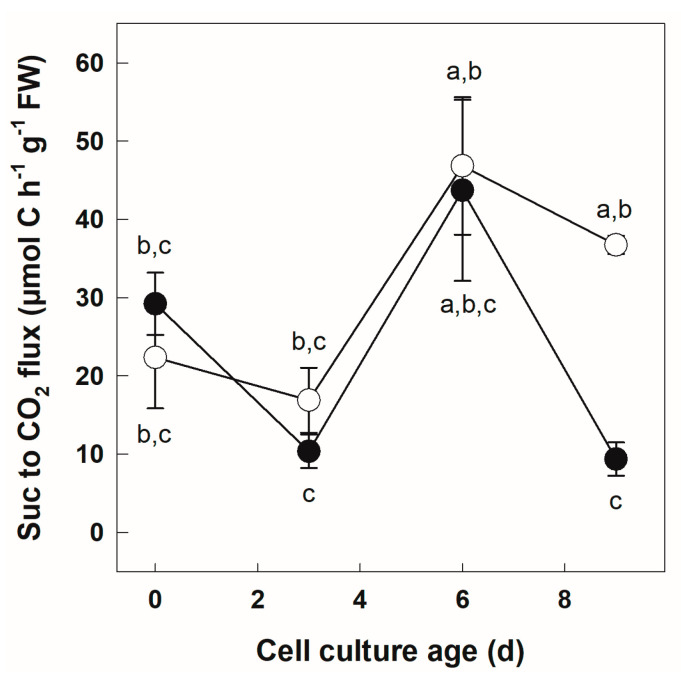
C flux between Suc and CO_2_ as a function of culture time in +Pi and −Pi potato cells. Cells growing under two different nutritional Pi regimes were incubated with [U-^14^C]Suc for 6 h. ^14^CO_2_ produced by the cells was trapped in KOH and quantified by scintillation counting. The molar C flux between Suc and CO_2_ was calculated using recovered radioactivity in the KOH trap and the specific radioactivity of fed [U-^14^C]Suc. Data points represent mean ± SD (*n* = 3 biological replicates). Different letters next to symbols (●: +Pi; ○: −Pi) indicate significant statistical differences (*p* < 0.05, Fishers LSD test).

Next, we quantified the molar C flux between Suc and CO_2_ using the specific radioactivity of fed [U-^14^C]Suc (Figure 3).

The calculated C flux values followed a very similar pattern in +Pi and −Pi cells between Days 0 and 6. Initially, the C flux between Suc and CO_2_ was between 20 and 30 µmol C h*^−^*^1^ g*^−^*^1^ FW. In both cultures, C flux between Suc and CO_2_ decreased slightly between Day 0 and Day 3. Under both Pi regimes, C flux increased transiently between Days 3 and 9 with a maximum value slightly over 40 µmol C h*^−^*^1^ g*^−^*^1^ FW at Day 6. However, the only significant difference between the +Pi and −Pi cells was observed at Day 9, with a C flux between Suc and CO_2_ of more than three times higher in the Pi-deficient than in the Pi-sufficient culture (Figure 3, Fishers LSD test, *p* < 0.05).

### 3.3. Labeling of +Pi and −Pi Cells with NaH^14^CO_3_

**Figure 4:**
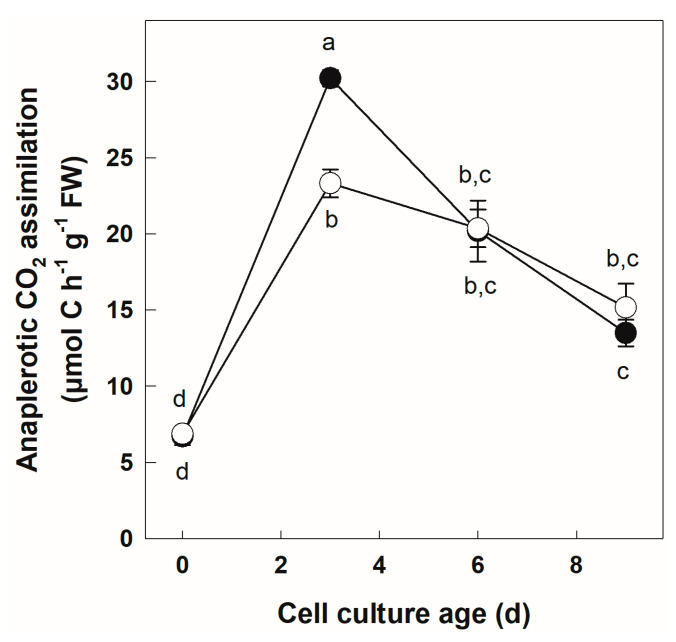
Anaplerotic C assimilation as a function of culture time in +Pi and −Pi potato cells. Cells growing under two different nutritional Pi regimes were incubated for 10 min with NaH^14^CO_3_. Labeling was stopped by addition of an acidic ethanol solution, and unincorporated ^14^CO_2_ was recovered in a KOH trap. ^14^CO_2_ incorporated in the acid soluble fraction was quantified by scintillation counting. The flux of anaplerotic C assimilation was determined from the specific radioactivity of fed NaH^14^CO_3_. Data points represent mean ± SD (*n* = 3 biological replicates). Different letters next to symbols (●: +Pi; ○: −Pi) indicate significant statistical differences (*p* < 0.05, Fishers LSD test).

Cells growing in Pi-sufficient and Pi-deficient conditions were incubated with NaH^14^CO_3_. At the end of the incubation, metabolism was stopped by the injection of an acidic ethanol solution into the culture medium. This killed the cells and volatilized any unincorporated labels. We determined acid-stable soluble radioactivity, which corresponds to the incorporation of the label in ethanol-soluble metabolites by anaplerotic pathways. The results of this experiment (Figure 4) showed, again, very similar trends in +Pi and −Pi cells. In an initial phase between Day 0 and Day 3, there was a large and significant surge in anaplerotic C assimilation, which increased about four and five times, respectively, in −Pi and +Pi cells. A significant difference was observed at Day 3 between Pi-sufficient and -deficient conditions (Fisher*’*s LSD test, *p* < 0.05). However, beyond this time point, anaplerotic C assimilation decreased steadily under both Pi regimes, and no significant difference was detected between the two cultures.

### 3.4. Analysis of the Interaction between Cell Culture Age and C Fluxes

The interaction between cell culture age and the various metabolic parameters measured during tracer experiments was performed using two-way ANOVA with a significance level at *p* < 0.05 (Table 1). The data indicate a significant effect of cell culture age on all measured parameters except for the % of [U-^14^C]Suc metabolized to cationic compounds. The effect of cell culture age was particularly strong in the cases of C flux between Suc and CO_2_ and anaplerotic CO_2_ assimilation (*p* < 0.001). Interestingly, no significant correlation could be observed between the Pi regime and the various metabolic parameters. The only significant interaction between phosphate regime and cell culture age was observed for anaplerotic CO_2_ fixation (Table 1).

## 4. Discussion

There is compelling evidence that nutritional Pi deficiency impacts plant life at the developmental, physiological and metabolic levels [2,3,19,40,41,42,43]. Low Pi availability results in a general decrease in free cellular Pi, negatively impacting the pool size of adenylates and phosphate esters [24,44,45,46]. Pi-deprived roots also generate and release organic anions such as citrate or malate [2,47,48,49,50]. Secretion of these organic acids has sometimes been linked to enhanced activity of carbon metabolism enzymes, in particular PEPC [2,47,51,52]. Another impact of Pi limitation on metabolism can be found at the level of the expression of genes encoding carbon metabolism enzymes and the resulting changes in protein steady-state level or enzyme activity [24,28,45,53]. Notably, Pi-deprived cells use inborn flexibility in their metabolism and rely on the induction of PPi-dependent enzymes or adenylate-independent enzymes to provide alternative routes for carbon flow, thereby allowing the maintenance of respiratory metabolism under stress [13,19,54,55].

One specific aspect of the Pi-deficiency response in plants that remains less characterized is how changes in enzyme activity or metabolite levels observed under stress possibly relate to changes in metabolic fluxes. Understanding the modulation of metabolic fluxes during environmental stress is essential to have a broad understanding of how plants adapt to these conditions [56,57,58,59,60]. In recent years, several studies on the effect of mineral nutrition on metabolic fluxes have been carried out in plants [25,61,62,63,64]. In an investigation of the response of potato cell cultures to Pi deprivation, we showed that the activity of a large number of C metabolism enzymes was increased by Pi deprivation [24]. In the same experimental system, we documented the maintenance of futile cycles between the pools of hexoses and hexose phosphates. Such cycles could consume ATP, which is a scarce commodity in cells at low internal Pi. These findings also imply that a maintenance of ATP generating pathways is taking place under Pi starvation. In order to further investigate these issues, we conducted experiments to measure important C fluxes under Pi starvation.

### 4.1. Sugar Uptake Is Maintained in Potato Cell Cultures Subjected to Pi Deficiency

Heterotrophic cell cultures relied on Suc as a C source. Due to the activity of the cell wall bound invertase, Suc may be hydrolyzed to glucose (Glc) and fructose (Fru) in the cell periplasm [65]. Transport of Suc, Glc and Fru from the medium by suspension cell cultures has been documented in various systems [66,67,68,69]. Sugar acquisition from the medium is likely due to specific active transporters, although endocytosis could also contribute to the process [66,70,71]. In +Pi and −Pi potato cells, the uptake of fed ^14^C sugars was quantified over culture time (Figure 1). For the first 9 days, both cultures showed similar uptake rates, indicating that Pi deficiency did not negatively impact sugar acquisition from the medium. This may seem surprising given the facts that (i) sugar acquisition relies, at least partially, on ATP [68,69], and (ii) ATP levels were drastically reduced in Pi-deficient cells [24]. Nevertheless, in the same material, Adenylate Energy Charge ratio and O_2_ uptake levels were similar to the Pi-replete cultures [24]. These results are thus consistent with the notion that, while Pi deficiency provokes a large perturbation in several metabolic processes, the expenditure of ATP necessary for the C uptake is maintained at a steady level for the first 9 days of stress. Nevertheless, at Day 12, a striking difference in sugar acquisition appeared between +Pi and −Pi cells. Pi-deficient cells maintained significant sugar transport (and presumably ATP costs associated with it), whereas very low uptake activity was found in the Pi replete culture. At this time point, dry weight accumulation reached plateau in the Pi-sufficient culture [24]. It is thus possible that culture entry in a stationary phase coincides with a strong decrease in the expression of genes involved in the nutrient uptake and primary metabolism as documented for Arabidopsis [72]. This could possibly shut down the use of sugars by cells reaching the stationary phase.

### 4.2. Sugar Catabolic Fluxes Are Similar in +Pi and −Pi Cells

Between days 0 and 9 of culture, uptaken sugars were used for catabolism through glycolysis and the Krebs cycle as evidenced by the production of ^14^CO_2_, as well as ^14^C-labeled anionic and cationic compounds (Figure 2 and Figure 3). Importantly, no qualitative difference in the fate of metabolized radioactivity could be observed between +Pi and −Pi cells (Figure 2), indicating the maintenance of organic and amino acid production. It is nevertheless very likely that specific pools of metabolites in these families of molecules were affected, as reported before [2,73,74]. The C flux from Suc to CO_2_ was quantified (Figure 3). The only difference between the two Pi regimes was found in cells at Day 9, for which −Pi cells maintained a higher C flux compared to +Pi cells. It should be pointed out that this severe reduction in the uptake for +Pi cells was not caused by a lack of cell viability towards the end of the culture cycle. Indeed, we have previously shown an increase in fresh weight and viable cell numbers between Days 9 and 12 for +Pi cells [24]. Although cell counts and fresh weight increase between Days 9 and 12, dry weight accumulation by the cultures already reaches its maximum [24]. Thus, it is possible that the sharp decrease in Suc to the CO_2_ flux found in +Pi cells between Days 6 and 9 is linked to the onset of the above-mentioned stationary phase changes associated with gene expression [72]. Accordingly, sugar uptake at Day 9 was below that of −Pi cells (Figure 1). An alternative reason for the lower Suc to CO_2_ flux at Day 9 in +Pi compared to −Pi cells resides in a change in the source of C for respiratory metabolism. For example, the mobilization of a stored Suc pool or the onset of starch reserves hydrolysis dilutes the pool of the ^14^C-label used to fuel glycolysis and respiration. The latter possibility, however, seems unlikely, since the Pi status is known to promote starch accumulation rather than its degradation [75,76,77].

The catabolism of fed [U-^14^C]Suc was evaluated in a qualitative and quantitative manner (Figure 2 and Figure 3, respectively). With the exception of C flux measured at Day 9 discussed above, the most striking result is the great similarity in the use of Suc to produce CO_2_, neutral, anionic and cationic compounds between +Pi and −Pi cells (Figure 2). Cell culture age was the main factor affecting the use of the supplied respiratory substrate (Table 1). These data indicate that, in both nutritional regimes, there is a relative stability in the allocation of metabolized C between CO_2_, neutral, anionic and cationic fractions. This may seem surprising since the two cell cultures presented large differences in growth curves [24]. The data are, however, consistent with the fact that similar O_2_ uptake and Adenylate Energy Charge values were recorded in Pi-sufficient and -deficient cultures [24]. These results are reminiscent of the earlier findings on the characterization of metabolic fluxes in heterotrophic tomato cell cultures subjected to various Glc feeding regimes [78]. This study demonstrated a marked stability in central C fluxes (glycolysis, the pentose phosphate pathway and the Krebs cycle) despite large differences in the uptake of Glc used as a C source or sampling at different stages of the culture cycle. The conclusion of these investigations was that, under the studied conditions, flux changes were mainly occurring at the level of anabolic pathways [78]. The stability of central C fluxes was seen as a key component of the architecture of plant cell metabolism. Our results are consistent with the view that Suc catabolic fluxes also remain relatively stable in cells experiencing Pi deprivation.

### 4.3. Metabolism of NaH^14^CO_3_ in +Pi and −Pi Cells Allows a Better Understanding of the Role of PEPC in the Stability of Anaplerotic C Flux under Pi Deficiency

Induction of PEPC activity in plants subjected to Pi deficiency has been documented in several systems [20,21,22,23,24]. In Pi-deficient potato cell cultures, significant concomitant increases in PEPC activity and PEPC protein have been documented [24]. The activity of PEPC results in the replenishment of C skeletons to the Krebs cycle allowing the sustainability of respiration and the production of organic acids [26]. In the present study, the incorporation of inorganic carbon into ethanol-soluble organic intermediates was examined using labeling with NaH^14^CO_3_. This measure reflects flux due to the in vivo operation of anaplerotic enzymes such as PEPC [38,79,80]. Cells incubated with NaH^14^CO_3_ readily incorporated the tracer in an acid-soluble fraction (Figure 4). The pattern of incorporation displayed similar trends in +Pi and −Pi cells. It was at its lowest in cells at Day 0. The highest flux values were obtained at Day 3 of the culture, indicating a strong anaplerotic C fixation activity early in the cell culture. In the +Pi cell culture, it would be reasonable to expect this result since Day 3 of the culture coincides with the beginning of the log phase of growth [24]. At this culture stage, there is an increased demand for anabolic reactions, as documented for the flux though PEPC in tomato cells [78]. In −Pi cells, an increase in anaplerotic assimilation of HCO_3_^−^ between Days 0 and 3 may be partly linked to a demand to sustain growth since an increase in dry weight was measurable between Days 0 and 3 [24]. After this initial period, the anaplerotic flux declined slowly and steadily as a function of culture time in both +Pi and −Pi cultures. Similar decreased values were observed in aging tomato cell cultures [78]. When comparing +Pi and −Pi cells, the flux values were only significantly different at Day 3, with a higher value in +Pi cells. It was found, in a previous study conducted on Arabidopsis, that cell cultures exposed to differing levels of nutritional Pi responded by a redistribution of C fluxes [25]. Notably, in low-Pi conditions, there was evidence for a greater degradation of Glc used as tracer in the plastid as well as a higher flux through PEPC [25]. While these differences could be due to different experimental approaches (pulse labeling vs. steady-state flux analysis) and plant material (potato is mycotrophic, whereas Arabidopsis is not), they could also reveal diverse metabolic flux responses. This points out the necessity to expand investigations on the behavior of metabolic fluxes under Pi deficiency.

It is interesting to compare this in vivo flux measurement with extractible PEPC activity values obtained in the same material [24]. As mentioned above, we previously documented induction of PEPC activity and protein levels by Pi deficiency. In contrast, the present study shows that the flux though PEPC changes very little between +Pi and −Pi conditions. To resolve this apparent discrepancy, it is crucial to consider that metabolites are an important component of the regulation of metabolic fluxes [81]. In vivo enzyme rates, such as that of PEPC in the present study, are influenced by substrate (phospho*enol*pyruvate) abundance [82] and by effector concentrations (e.g., glucose-6P (G6P), an activator and malate, an inhibitor) [83]. In vivo, metabolic flux values are also influenced by enzyme amounts, generally quantifiable by extractible activity and by post-translational regulation. In the case of Pi-deficiency-induced phosphorylation of PEPC, it was shown that this modification brings about an increased sensitivity to G6P activation and a lower sensitivity to inhibition by malate [13]. Pi deficiency generally leads to a decrease in G6P and an increase in malate [2,24,46,47]. Thus, our study supports the view that the previously documented induction of PEPC in Pi-deficient cells [24] reflects a need to adjust the amount of the active enzyme in order to maintain the relative stability in the anaplerotic C flux in conditions of low G6P and high malate.

## 5. Conclusions

The present research provided some insights in a previously understudied area of plant adaptation to nutritional Pi limitation. Here, we showed that in Pi-limited potato cell cultures, there is a general stability in important C fluxes such as sugar uptake from the medium, carbohydrate catabolism to fuel respiration and anaplerotic C fixation. Thus, despite the fact that large changes in metabolites and enzyme levels occur in this system [24], cells are able to maintain important catabolic and anabolic fluxes. We interpret the previously documented changes in enzyme levels under low Pi as a strategy to maintain the stability of cellular C fluxes.

## Figures and Tables

**Table 1:** Analysis of variance (ANOVA) showing the fixed effects on cell culture age and phosphate regime for metabolic parameters. The significance level is noted as follows: *, *p* < 0.05; **, *p* < 0.01; ***, *p* < 0.001.

Parameter	Cell Culture Age		Phosphate Regime		Phosphate Regime x Cell Culture Age	
	*F*	*p*	*F*	*p*	*F*	*p*
C uptake (μmol C h^−1^·g^−1^ FW)	6.22	0.002 **	2.15	0.157	1.43	0.260
% CO_2_ in metabolized [U-^14^C]Suc	4.92	0.013 *	1.33	0.2667	0.849	0.4874
% Neutral fraction in metabolized [U-^14^C]Suc	4.14	0.023 *	0.74	0.401	0.214	0.8850
% Anionic fraction in metabolized [U-^14^C]Suc	5.46	0.008 **	2.60	0.126	0.41	0.743
% Cationic fraction in metabolized [U-^14^C]Suc	0.14	0.932	0.030	0.865	0.188	0.903
Suc to CO_2_ flux (μmol C h^−1^ g^−1^ FW)	11.16	3.37 × 10^−4^ ***	3.22	0.0916	2.94	0.064
Anaplerotic CO_2_ assimilation (μmol C h^−1^ g^−1^ FW)	83.9	5.25 × 10^−10^ ***	3.03	0.100	5.25	0.010 *

## Data Availability

The biological material and the data presented in this study are available from the corresponding author upon reasonable request.
